# Cytotoxic Effect and TLC Bioautography-Guided Approach to Detect Health Properties of Amazonian* Hedyosmum sprucei* Essential Oil

**DOI:** 10.1155/2016/1638342

**Published:** 2016-03-28

**Authors:** Alessandra Guerrini, Gianni Sacchetti, Alessandro Grandini, Antonella Spagnoletti, Mercedes Asanza, Laura Scalvenzi

**Affiliations:** ^1^Department of Life Sciences and Biotechnology (SVeB), University of Ferrara, Corso Ercole I d'Este 32, 44121 Ferrara, Italy; ^2^Universidad Estatal Amazónica, Via Napo Km 2 1/2 Paso Lateral, 160150 Puyo, Ecuador

## Abstract

Bioautography has been used as rapid and easy strategy to detect and identify bioactive fractions/molecules in the never before investigated* Hedyosmum sprucei* Solms (Chloranthaceae) essential oil (EO). The antioxidant activity, performed through DPPH bioautographic assay and spectrophotometric evaluations (IC_50_ = 230 ± 10 *µ*g/mL), seemed to be mainly due to *α*-cadinol and *α*-muurolol. (HP)TLC bioautography, focused on antimicrobial capacities, pointed out *α*-cadinol, *α*-muurolol, *τ*-muurolol, caryophyllene oxide, and methyleugenol as the most effective compounds against* Staphylococcus aureus*, considered as testing strain. Moreover, the microdilution method, assessed among a wide panel of microorganisms, revealed* Listeria grayi* and* Staphylococcus aureus* as the most sensitive among human tested strains and* Clavibacter michiganensis* among phytopathogens. GC-MS chemical profile showed that bioactive molecules represented only a small quantity of the whole EO: germacrene D (23.16%), *β*-caryophyllene (15.53%), *δ*-cadinene (5.50%), *α*-copaene (5.08%), and *α*-phellandrene (3.48%) were the main compounds, highlighting an uncommon composition among the genus* Hedyosmum*. Finally,* H. sprucei* EO was checked for cytotoxic potential against A549 (lung cancer) and MCF-7 (breast cancer) cell lines showing promising cytotoxic effects against both cell lines after 48 h (IC_50_ A549 = 44.05 ± 2.35 *µ*g/mL; IC_50_ MCF-7 = 32.76 ± 4.92 *µ*g/mL) and 72 h (IC_50_ A549 = 43.55 ± 2.80 *µ*g/mL; IC_50_ MCF-7 = 33.64 ± 0.43 *µ*g/mL).

## 1. Introduction

Plants are sources of huge number of bioactive lead/scaffolds with therapeutic and nutraceutical importance. Bioautography is a fast technique that allows us to identify and isolate bioactive fractions/compounds in never previously studied plant extracts by employing a suitable chromatographic process followed by a biological detection system [1, 2].

*Hedyosmum sprucei* Solms (Chloranthaceae) is an aromatic shrub growing on the eastern versants of the tropical Andes of Ecuador and Peru, in disturbed and undisturbed sites, such as secondary rainforest and streamsides, as well as primary habitats such as cloud forest. The species is found at 500–2000 m.a.s.l.* H. sprucei*, known in the Pastaza region of Ecuador as “sacha limón panga,” “sacha limón caspa,” or “hoja de monte,” has leaves with a pleasant fragrance similar to a clove-like scent that leaves the mouth slightly numb if chewed [3]. Moreover, all broken parts of the plant emit the typical pungent smell, similar to pepper, lemon, and anise. That odor gives the name to the genus, which has Greek origin:* hedy* (pleasant) plus* osmium* (smelling) [4]. A common feature of the genus is the presence of secretory cells in the leaves and stems [5]. The genus* Hedyosmum* belongs to the Chloranthaceae family, considered primitive among Angiospermae, including four genera and 75 species.* Hedyosmum* has 40–50 species of shrubs or small trees, in low and high mountain rain forests, with greatest diversity in the Andes of South America and a secondary center of diversity in the mountains of southern Mexico and Central America; a single species,* H. asiaticum*, is native to South-East Asia. The genus is characterized by dentate and opposite leaves, with petioles sheathed on the base, and by unisexual diclinous flowers.

To the best of our knowledge no studies about* H. sprucei* phytochemistry have been previously reported. However, chemical composition of* Hedyosmum* species was performed on* H. arborescens* [6],* H. angustifolium*,* H. scabrum* [7],* H. brasiliense* [5, 7],* H. bonplandianum*,* H. costaricensis*,* H. mexicanum* [5, 8], and* H. columbianum* [9].

In light of the ethnomedical uses that report the leaf infusion of* H. sprucei* employed by Natives as internal and topical preparations to treat snakebites [3], this paper intends to be the first report based on chemical characterization and biological properties evaluation (antioxidant, antibacterial, and cytotoxic) about* H. sprucei* derivative, in order to investigate a possible new food and/or functional health ingredient profile from that plant, using the fast bioautography to detect the main active fractions/molecules. The aromatic quality of the plant drove the research towards the study of the essential oil. In recent decades, essential oils have become acclaimed by consumers for their use as biologically active natural ingredients, in a wide range of products: food, cosmetics, beverage, pharmaceutics, and pesticides. Moreover, the essential oils are plant extracts which benefit from both the increasing interest of consumers as functional natural compounds and the consequent interest of the research of food and health natural molecules [10].

## 2. Materials and Methods

### 2.1. Plant Material

To determine the taxonomic identification, two specimens of* H. sprucei* Solms were collected for morphological and histological evaluations from wild plants in the Amazonian region of Pastaza, Ecuador. The first specimen, collected in March 2013 before the flowering period, represented the crude drug, while the second one, collected in full anthesis in April 2013, was employed for authentication purposes. Voucher specimens were certified by Dr. David Neill and deposited at the Herbarium ECUAMZ of the Amazonian State University (UEA) in Ecuador (voucher specimens: D. Neill 17672a, 17672b).

### 2.2. Isolation of Essential Oil

The essential oil was obtained from fresh aerial parts of the plant, before the flowering period, by 2 h hydrodistillation in a stainless steel distiller equipped with a Clevenger apparatus. Essential oil yield (0.03%) was calculated on a moisture-free basis and determined as average of three distinct distillations. The oil was dried over anhydrous sodium sulphate and stored in sealed amber vials at 4°C.

### 2.3. GC and GC/MS Analysis

Essential oil was analyzed and the relative peak areas for individual compounds were averaged. For the analysis a ThermoQuest GC-Trace gas-chromatograph equipped with a FID detector and a Varian FactorFour VF-5ms poly-5% phenyl-95%-dimethylsiloxane column (i.d., 0.25 mm; length, 30 m; film thickness, 0.15 *µ*m) were used. Operating conditions were as follows: injector temperature 300°C, FID temperature 300°C, carrier (Helium) flow rate 1 mL/min, and split ratio 1 : 50. The initial oven temperature was 55°C and then rose to 100°C at a rate of 1°C/min, then rose to 250°C at a rate of 5°C/min, and then kept constant at 250°C for 15 min. One microliter for each replicate was dissolved in CH_2_Cl_2_ and injected. The oil percentage composition was computed by the normalization method from the GC peak areas, without using correction factors. The chemical characterization of essential oil compounds was performed by a Varian GC-3800 gas-chromatograph equipped with a Varian MS-4000 mass spectrometer using electron impact and hooked to NIST library. The conditions were the same as described for GC analysis and also the same column was used. The mass spectroscopy conditions were as follows: ionization voltage, 70 eV; emission current, 10 *µ*Amp; scan rate, 1 scan/s; mass range, 29–400 Da; trap temperature, 150°C; and transfer line temperature, 300°C. The essential oil compounds were characterized by comparing their relative retention time, KI, and the MS fragmentation pattern with those of other known essential oils and with pure compounds and by matching the MS fragmentations patterns and retention indices with the above-mentioned mass spectra libraries and with those in the literature [11]. The Kovats index of the components was determined adding C_8_–C_32_ *n*-alkanes (Sigma-Aldrich) to the essential oil before injection in the GC-MS equipment and analyzed under the same conditions reported above [12].

### 2.4. Biological Activities of* H. sprucei* Essential Oil

The* H. sprucei* essential oil has been subjected to antioxidant, antibacterial, and cytotoxic activities, applying the methods described below. All the bioactivities were carried out comparing data with those obtained with appropriate pure synthetic compounds, with the aim of having positive control references with single chemicals. Data reported are the average of three measurements of three independent experiments.

#### 2.4.1. Radical Scavenging and Antibacterial HPTLC Bioautography

High-performance-thin-layer-chromatography (HPTLC) bioautography was performed in order to localize, separate, and identify those compounds responsible for the radical scavenging and antibacterial activities of the essential oil, according to previously described methods [13]. In particular, in light of the knowledge acquired by the research team in the last few years, the identification of possible antibacterial fractions of* H. sprucei* essential oil was carried out on the human pathogen* Staphylococcus aureus* (ATCC 29230), because of its high performant features on (HP)TLC plate assay.

Three aliquots (6, 30, and 30 *μ*L) of the ethanol solution of* H. sprucei* essential oil (110 mg/mL) were applied to silica gel (HP)TLC plates (CAMAG, Muttenz, Switzerland) as 10 mm wide bands with Linomat IV (CAMAG, Muttenz, Switzerland) and eluted for 8 cm in a chromatographic chamber with a solvent solution characterized by toluene/ethyl acetate/petroleum ether 93/7/20. After development and complete drying, TLC plates were cut and separated into three single chromatograms. The plate with the 6 *μ*L aliquot was sprayed with the ethanol solution of DPPH radical (20 mg/100 mL) to determine the antioxidant fractions. The active constituents were recognized as yellow areas on a violet background.

A chromatogram plate with the 30 *μ*L aliquot of essential oil was disposed in Petri dishes together with a proper agarized medium previously added with the bacterial inoculum (10^5^ CFU/50 mL) and 0.25% of a 2,3,5-triphenyl-tetrazolium chloride (Sigma-Aldrich, St. Louis, USA) water solution (20 mg/mL), as growth indicator. Petri dishes were incubated overnight at 37°C. Antibacterial chemicals appeared as clear yellow spots against a red colored background.

Finally, the last chromatogram plate was used to isolate and identify the active compounds: TLC areas showing positive radical scavenging and antibacterial activity were removed, then extracted with methanol, and analyzed by GC-FID and GC-MS.

#### 2.4.2. Quantitative Spectrophotometric DPPH Assay

An essential oil solution of* H. sprucei* was prepared by dissolving 10 *µ*L of essential oil with 900 *µ*L of ethanol; further dilutions were set up in order to obtain different concentrations (0.8–6.67 × 10^−4^ *μ*L/mL). An aliquot (2.9 mL) of ethanol solution of DPPH (4 mg/100 mL) was added to essential oil solution. After 30 min incubation at 200 rpm, in the dark at room temperature, the mixture was placed in a UV-Vis spectrophotometer (Thermo-Spectronic Helios *γ*, Cambridge, UK) and the absorbance was read in triplicate against a blank at 517 nm. The inhibition of DPPH in percent was determined by the following formula: *I*_DPPH_% = [1 − (*A*_1_/*A*_2_)]  × 100, where *A*_1_ is the DPPH absorbance with essential oil and *A*_2_ without essential oil. As positive control, commercial thymol (Sigma-Aldrich, St. Louis, USA) was used, in light of its well-known antioxidant properties [14]. Essential oil antioxidant activity was expressed as IC_50_ (concentration providing DPPH 50% inhibition), determined from inhibition curves obtained by plotting inhibition percentage against essential oil concentration. All experiments were performed in triplicate and values were reported as mean ± SD (standard deviation).

#### 2.4.3. Quantitative Antibacterial Activity

The antibacterial activity of* H. sprucei* essential oil was quantified by microdilution method, using 96-well microtiter plates [15], in order to determine MIC (Minimum Inhibitory Concentration), against the following human pathogen bacterial strains: the Gram negative* Escherichia coli* (ATCC 4350) and* Pseudomonas aeruginosa* (ATCC 27853) and the Gram positive* Listeria grayi* (DSM 20601) and* Staphylococcus aureus* (ATCC 29230). Moreover, bioactivity against phytopathogen bacteria was checked versus the Gram negative* Agrobacterium tumefaciens* (DSM 30207),* Agrobacterium vitis* (DSM 6583),* Pseudomonas syringae* pv.* syringae* (DSM 10604), and Gram positive* Clavibacter michiganensis* subsp.* nebraskensis* (DSM 20400).

For all the bacterial strains the antibacterial activity was determined in terms of MIC, checked through the microdilution method. Gram negative (*A. tumefaciens* and* A. vitis*) bacterial cultures were incubated in Nutrient Broth (Sigma-Aldrich, St. Louis, USA), at 37°C overnight, and the remaining strains in Tryptic Soy Broth (OXOID Ltd., Hampshire, UK). Sterile medium (100 *μ*L) was pipetted into all wells together with 100 *μ*L of serial dilutions of* H. sprucei* essential oil previously dissolved in ethanol (100 mg/mL). Serial dilutions were prepared in order to obtain concentration ranges from 0.03 *μ*L/mL to 2 *μ*L/mL. One hundred *μ*L of bacterial culture standardized to 2 × 10^7^ CFU/mL was added to the wells and incubated at 37°C for 6 h and at 26°C for 24 h, respectively, for human and phytopathogens. After incubation, 40 *μ*L of a 2,3,5-triphenyl-tetrazolium chloride (Sigma-Aldrich, St. Louis, USA) water solution (20 mg/mL) was added to each well and then incubated. After 30 min, microbial growth was evaluated by microplate reader (680XR, Bio Rad, Laboratories, Inc., Hercules, CA, USA) at 615 nm. Commercial thymol, due to its known antimicrobial properties [16], was considered as positive control, at the same concentrations of* H. sprucei* essential oil. Sterile medium served as growth control.

#### 2.4.4. Cytotoxic Activity

A549 (human lung cancer cell line) and MCF-7 (human breast cancer cell line) were purchased from “Istituto Zooprofilattico Sperimentale della Lombardia e dell'Emilia-Romagna,” Brescia, Italy. The cell lines were grown in Dulbecco's modified Eagle's medium supplemented with 10% fetal bovine serum (FBS), 100 U/mL penicillin/streptomycin, and 2 mM L-glutamine. The cell lines were grown in 75 cm^2^ flasks in a humidified 5% CO_2_-95% air atmosphere at 37°C until 80% confluence.

Cytotoxic activity was determined by MTT colorimetric assay [17] as reflected by the activity of succinate dehydrogenase. Briefly, cells were seeded in 96-well plates at a density of 2 × 10^4^ cells/well in 200 *µ*L DMEM complete medium and allowed 24 h for attachment. Then the culture medium was replaced with 200 *µ*L medium containing different concentrations (from 1 to 100 *µ*g/mL) of* H. sprucei* essential oil. Similar assay was carried out with *β*-caryophyllene standard, in order to evaluate its possible cytotoxic activity considering the high amount in the essential oil. Control culture was exposed to only vehicle (medium containing 2% FBS). After 24, 48, and 72 h, the culture medium was removed and washed with PBS (phosphate-buffered saline) twice, and 20 *μ*L of MTT (5 mg/mL in PBS) was added in each well and the plates were incubated for 4 h at 37°C. The medium was removed and replaced with 100 *μ*L dimethyl sulphoxide to dissolve the formazan crystals. The extent of MTT reduction was measured spectrophotometrically at 570 nm using a microplate reader (680XR, Bio Rad, Laboratories, Inc., Hercules, CA, USA). The cytotoxic activity was expressed as the concentration of sample that inhibited 50% of cell growth (IC_50_).

### 2.5. Statistical Analysis

The experiments were performed in triplicate. IC_50_ values of antioxidant and antiproliferative activities were determined by logarithmic regression curves with 95% confident limits. Relative standard deviations and statistical significance (Student's *t*-test; *p* ≤ 0.05) were calculated using software STATISTICA 6.0 (StatSoft Italia srl).

## 3. Results and Discussion

### 3.1. Chemical Composition of Essential Oil

The chemical composition of* H. sprucei* essential oil is reported in Table 1. Fifty-eight compounds were identified, corresponding to 98.67% of the total. Sesquiterpenes represent 88.57% and monoterpenes 10.10% of all the compounds detected and identified. The 17.06% of the total is oxygenated. Among the sesquiterpenes the most abundant were germacrene D (23.16%), *β*-caryophyllene (15.53%), *α*-cadinene (5.50%), and *α*-copaene (5.08%); among the monoterpenes *α*-phellandrene was the most abundant (3.48%). Comparing our data to that provided by the related literature on different* Hedyosmum* species, an interesting result is the high sesquiterpene abundance which characterized the* H. sprucei* essential oil as compared to that of* H. brasiliense* [5],* H. angustifolium*,* H. scabrum* [7],* H. arborescens* [6],* H. bonplandianum,* and* H. mexicanum* [8], mainly typified by monoterpenes. The only* Hedyosmum* species which is known for its sesquiterpene-rich essential oil is* H. costaricense* [8]. Apart from this, the sesquiterpenes *α*-cadinene and *α*-copaene, found in moderate concentration in* H. sprucei*, did not occur in* H. arborescens*,* H. mexicanum*,* H. scabrum*, and* H. angustifolium*, while they were reported in* H. brasiliense*,* H. costaricense,* and* H. bonplandianum* in amount of less than 1%. The sesquiterpenes *α*-cadinol and *α*-muurolene were previously observed just in* H. costaricense* and the 1-epi-cubenol was previously observed just in* H. brasiliense*; furthermore, the latter species were the only ones to contain methyleugenol. Apart from* H. sprucei*, none of the species taken into account held compounds as *α*-muurolol, *γ*-muurolene, 1,10-di-epi-cubenol, and *τ*-cadinol. These results, as a first report about the* H. sprucei* essential oil, represent a further contribution to the phytochemistry of the genus. However, the difference detected between* H. sprucei* essential oil and those of the other* Hedyosmum* species could be also related to the Amazonian origin of our samples. In fact, the high biodiversity of Amazonian region is known to drive the plant secondary metabolism to biosynthetic pathways which are particularly diversified in chemical structures [18].

### 3.2. Radical Scavenging and Antibacterial Bioautography

**Figure 1:**
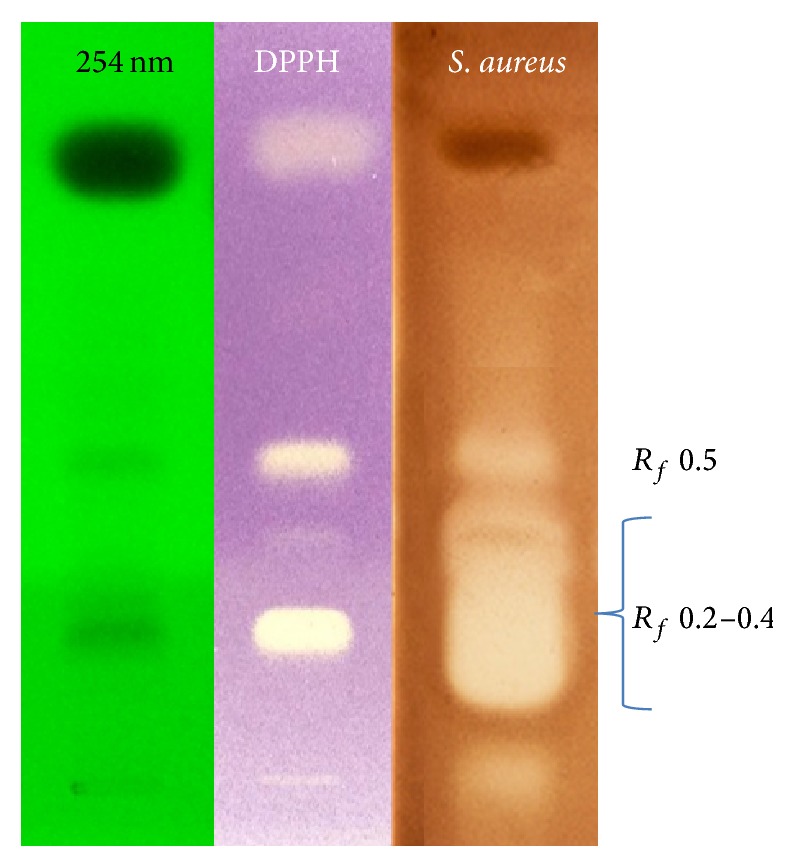
DPPH-radical scavenging and antibacterial (HP)TLC bioautographic assay. Active compounds corresponding to the different *R*_*f*_ are reported in Table 1.

The DPPH (HP)TLC bioautography showed the antiradical capacity of* H. sprucei* essential oil, with particular reference to *R*_*f*_ 0.3 and 0.5. Three different areas on the TLC evidenced antioxidant properties, in particular the fractions corresponding to *R*_*f*_ 0.3, 0.5, and 0.9 (Figure 1). *R*_*f*_ 0.3, mainly characterized by *α*-cadinol (70.56%) and *α*-muurolol (29.44%), as evidenced by GC-MS, seemed to be the most responsible for the bioactivity, given the most evident reactivity to the DPPH radical. *R*_*f*_ 0.5 characterized by methyleugenol (60.23%), caryophyllene oxide (23.48%), 1,8-cineole (6.84%), 1,10-di-epi-cubenol (4.12%), 1-epi-cubenol (2.85%), and humulene 1,2-epoxide (2.48%) showed an intermediate activity, while *R*_*f*_ 0.9, composed of germacrene D (40.28%), *β*-caryophyllene (23.14%), *δ*-cadinene (8.78%), *β*-elemene (6.16%), *α*-copaene (5.72%), *α*-caryophyllene (5.55%), *α*-muurolene (2.00%), *γ*-muurolene (2.28%), *δ*-elemene (1.95%), bicyclogermacrene (1.85%), germacrene B (1.17%), and *δ*-cubenene (1.13%), evidenced the lowest reaction intensity. Given the fact that these results represent the first report concerning* H. sprucei* essential oil, however, some considerations could be supported in light of the related literature about single compounds characterizing the isolated fractions. In fact, the important involvement of the compounds characterizing *R*_*f*_ 0.3, as *α*-cadinol and *α*-muurolol, can be evidenced in antioxidant properties [19], analogously caryophyllene oxide, eugenol, elemicin, and methyleugenol for the fraction with *R*_*f*_ 0.51 [20, 21], and germacrene D and caryophyllene for *R*_*f*_ 0.9 [22].

(HP)TLC bioautography on* S. aureus* (Figure 1) evidenced two different areas corresponding to the fractions *R*_*f*_ 0.2– *R*_*f*_ 0.4 and *R*_*f*_ 0.5. The TLC area, extending from *R*_*f*_ 0.2 to *R*_*f*_ 0.4, became the most bioactive as antibacterial, as evidenced by the highest intensity of the clear yellow spot with reference to the fraction at *R*_*f*_ 0.5. The fraction *R*_*f*_ 0.2– *R*_*f*_ 0.4 was mainly characterized by *α*-cadinol (33.99%), *α*-muurolol (13.72%), *τ*-muurolol (10.78%), selin-11-en-4-*α*-ol (9.33%), linalool (6.01%), trans-muurola-4(14),5-diene (3.80%), *τ*-cadinol (3.33%), eudesma-4(15),7-dien-1-*β*-ol (3.12%), *δ*-amorphene (2.57%), elemol (2.59%), *γ*-eudesmol (2.53%), and *β*-atlantol (2.38%) and *R*_*f*_ 0.5 by caryophyllene oxide (45.30%), methyleugenol (26.33%), 1,10-di-epi-cubenol (18.81%), and humulene 1,2-epoxide (7.77%).

Some of the antibacterials detected in* H. sprucei* essential oil were reported to be active as pure compounds: *τ*-muurolol, *τ*-cadinol, methyleugenol [23], linalool [23, 24], selin-11-en-4-*α*-ol, caryophyllene oxide, *α*-cadinol, *α*-muurolol [25], and elemol [26]. *τ*-cadinol, revealed in fraction *R*_*f*_ 0.2– *R*_*f*_ 0.4, is known to exhibit bacteriolytic properties toward* S. aureus* [27], since it interacted with bacterial cell envelop, inducing cell lysis and subsequent death.

Methyleugenol, abundant in *R*_*f*_ 0.5 fraction, may be responsible for the detected antibacterial activity of* H. sprucei* essential oil, since the pure compound is pointed out as antimicrobial agent by related literature [28]. Moreover, related studies report this molecule that is generally used as flavor in food like jellies, baked goods, nonalcoholic beverages, chewing gum, candy, and ice cream, giving a spicy ginger-like undertone.

To the best of our knowledge, evidences are reported in the literature of antimicrobial activity of the following remaining compounds, as phytocomplex constituents: *δ*-amorphene [29], 1,10-di-epi-cubenol [30], and *γ*-eudesmol [26].

In conclusion, a lot of sesquiterpenes showed a potential activity. Future investigations on isolated compounds and their mixture could better highlight the lead molecules or synergic active complex.

### 3.3. Quantitative Determination of Radical Scavenging and Antibacterial Activity

The* H. sprucei* essential oil evidenced an interesting antioxidant activity if compared to standard positive control, not previously reported in the literature. In fact* H. sprucei* essential oil possessed a DPPH scavenging activity (IC_50_ = 230 ± 10 *μ*g/mL) about 30% better than thymol (IC_50_ = 318 ± 7 *μ*g/mL).

Antibacterial activity of* H. sprucei* essential oil was performed against Gram positive and negative human and plant pathogenic bacteria, through serial microdilution method for MIC (Minimum Inhibitory Concentration) determination. Results were compared to that obtained with thymol, taken as positive control, as suggested by the related literature [16] (Table 2). Among the human pathogen bacteria, the most sensitive strains were the Gram positive* L. grayi* and* S. aureus*, with the latter responsible for important nosocomial infections [5]. Gram negative bacteria were more resistant to treatments than Gram positive ones showing MIC even 50% higher.

Regarding plant pathogen bacteria, the MIC values were even more performing. In particular,* C. michiganensis* subsp.* nebraskensis* evidenced the lowest detected MIC (62 *μ*g/mL), 87% even better than positive control. Particular relevance should be given to the sensitivity of* P. syringae* pv.* syringae*, since the strain is responsible for important plant diseases related to equally important cultivations as kiwi (*Actinidia deliciosa* (A. Chev.) C. F. Liang & A. R. Ferguson), tobacco (*Nicotiana tabacum* L.), and mango (*Mangifera indica* L.).

### 3.4. Cytotoxic Activity

The cytotoxic activity was evaluated against lung adenocarcinoma cell line (A549) and breast adenocarcinoma cell line (MCF-7) expressed as IC_50_ values. The results showed that MCF-7 cell line exhibited higher sensitivity than A549 to treatments with* H. sprucei* essential oil. In particular, MCF-7 cells were affected with interesting growth inhibition with IC_50_ values of 32.76 ± 4.92 and 33.64 ± 0.43 *µ*g/mL after 48 and 72 h, respectively. According to the previous literature, values lower than 30 *µ*g/mL suggest a good chemopreventive potential for the extract [31]. In light of these premises,* H. sprucei* essential oil might be a promising source of active compound(s) for innovative therapeutic and/or preventive strategies against breast adenocarcinoma. On the other hand, less interesting were the data on A549 cell line with IC_50_ values between 44.05 ± 2.35 and 43.55 ± 2.80 *µ*g/mL. The selective toxicity of* H. sprucei* essential oil toward MCF-7 cells may be due to the sensitivity of the cell line to the active compounds in the phytocomplex or to tissue specific response [32]. It has been reported that terpenes such as germacrene D and *β*-caryophyllene exhibited cytotoxic activity against breast cancer cells; thus, the high content of these components in the* H. sprucei* essential oil may explain the cytotoxic activity toward MCF-7 [33–35]. To confirm this data, we have tested *β*-caryophyllene cytotoxicity at 48 h: IC_50_ values for MCF-7 cell line were 50.85 ± 7.35 *µ*g/mL and for A549 cell line 42.05 ± 0.50 *µ*g/mL. It has been also demonstrated that *β*-caryophyllene potentiated the cytotoxic activity of *α*-caryophyllene and other sesquiterpenes on MCF-7 cell line [34]. In light of this evidence, the high amount of sesquiterpenes, known for their anticancer activity, pointed out* H. sprucei* essential oil as significant potential source of pure compounds with promising anticancer activity [36].

## 4. Conclusion

In conclusion, this study characterized for the first time the components of* H. sprucei* essential oil and investigated its potential radical scavenging and antibacterial capacities by bioautography and quantitative methods. Moreover, it explored its cytotoxic properties. Preliminary results of this research were presented by our colleague and coauthor of the present paper, Spagnoletti et al., in 2014 [37]. The results highlighted its excellent radical scavenging activity and good antibacterial effects against Gram positive and negative bacteria including human and phytopathogens and promising cytotoxic activity against MCF-7 cell line. These results suggest that further investigations are required in order to evaluate the use* in vitro* and* in vivo* of* H. sprucei* essential oil, especially as food and dietary supplements aimed at oxidative stress prevention, representing an alternative to addition of synthetic antioxidant suspected of toxicity [38]. Moreover, it may be used in cosmetic products as antiage effective ingredient. Finally, possible uses of* H. sprucei* essential oil are viable for treatment of human and phytopathogenic bacterial diseases.

## Figures and Tables

**Table 1:** Chemical composition of *Hedyosmum sprucei *essential oil and characterization of active antioxidant and antibacterial compounds isolated, respectively, from (HP)TLC-DPPH^∙^ and (HP)TLC bioautography assays.

Number	% area^(a)^	Compounds	AI exp^(b)^	AI lett	% area^(a)^				
					Antioxidant fraction			Antibacterial fraction	
					*R*_*f*_ = 0.3	*R*_*f*_ = 0.5	*R*_*f*_ = 0.9	*R*_*f*_ = 0.2–0.4	*R*_*f*_ = 0.5
1	1.99	*α*-Pinene	929	932					
2	1.40	*β*-Pinene	973	974					
3	0.19	Myrcene	987	988					
4	3.48	*α*-Phellandrene	1005	1002					
5	0.27	p-Cymene	1021	1020					
6	0.50	Limonene	1025	1024					
7	0.31	*β*-Phellandrene	1026	1025					
8	0.29	1,8-Cineole	1027	1026		6.84			1.16
9	0.82	*trans*-Ocimene	1042	1044					
10	0.85	Linalool	1100	1095				6.01	
11	0.76	*δ*-Elemene	1340	1335			1.95		
12	0.98	*α*-Cubebene	1352	1345			1.13	0.59	
13	5.08	*α*-Copaene	1377	1374			5.72		
14	0.19	*β*-Bourbonene	1382	1387					
15	0.72	*β*-Cubebene	1387	1387				0.87	
16	1.87	*β*-Elemene	1388	1389			6.16		
17	1.86	Methyl eugenol	1401	1403		60.23			26.33
18	15.53	*β*-Caryophyllene	1412	1417			23.14		
19	0.37	*β*-Copaene	1424	1430					
20	3.47	*α*-Caryophyllene	1451	1452			5.55		
21	0.15	*allo*-Aromadendrene	1456	1458					
22	0.22	*cis*-Cadina-1(6),4-diene	1459	1461					
23	0.42	*γ*-Gurjunene	1470	1475					
24	1.64	*γ*-Muurolene	1474	1478			2.28		
25	23.16	Germacrene D	1479	1484			40.28		
26	0.55	*cis*-*β*-Guaiene	1484	1492					
27	0.31	*trans*-Muurola-4(14),5-diene	1487	1493				3.80	
28	0.15	*γ*-Amorphene	1489	1495					
39	1.71	Bicyclogermacrene	1491	1500			1.85		
30	1.08	*α*-Muurolene	1495	1500			2.00		
31	0.29	*trans*-*β*-Guaiene	1499	1502					
32	1.82	Germacrene A	1501	1508					
33	0.61	*δ*-Amorphene	1509	1511				2.57	
34	0.50	*γ*-Cadinene	1512	1513					
35	5.50	*δ*-Cadinene	1516	1522			8.78		
36	0.22	*trans*-Calamenene	1520	1522					
37	0.30	*trans*-Cadina-1(2)-4-diene	1530	1533					
38	0.18	*α*-Cadinene	1535	1537					
39	0.29	Elemol	1549	1548				2.59	
40	1.13	Germacrene B	1557	1559			1.17		
41	1.66	*trans*-Nerolidol	1562	1561				1.11	
42	0.17	Spathulenol	1577	1577				1.77	
43	0.91	Caryophyllene oxide	1581	1582		23.48			45.30
44	0.35	Humulene 1,2-epoxide	1609	1608		2.48			7.77
45	0.51	*β*-Atlantol	1617	1608				2.38	
46	0.58	1,10-di-epi-Cubenol	1623	1621		4.12			18.81
47	1.71	1-epi-Cubenol	1630	1629		2.85			0.62
48	0.42	*γ*-Eudesmol	1634	1632				2.53	
49	1.46	*τ*-Cadinol	1645	1638				3.33	
50	1.12	*τ*-Muurolol	1647	1642				10.78	
51	1.12	Cubenol	1650	1646					
52	1.65	*α*-Muurolol	1659	1652	29.44			13.72	
53	3.29	*α*-Cadinol	1662	1660	70.56			33.99	
54	1.35	Selin-11-en-4-*α*-ol	1645	1638				9.33	
55	0.22	Germacra-4(15),5,10(14)-trien-1-*α*-ol	1647	1642					
56	0.62	Eudesma-4(15),7-dien-1-*β*-ol	1690	1685				3.12	
57	0.19	*γ*-Costol	1694	1687					
58	0.18	Mint sulfide	1730	1745					

total	96.11								

^(a)^Relative peak areas were calculated by GC-FID. ^(b)^Arithmetic indices were calculated on a Varian VF-5ms column.

**Table 2:** Antibacterial activity of *H. sprucei* essential oil expressed as MIC (*μ*g/mL).

	*H. sprucei *essential oil	Thymol
	(*μ*g/mL)^(a)^	
Human pathogen bacteria		
Gram negative		
*Escherichia coli *ATCC 4350	>2000	2000
*Pseudomonas aeruginosa *ATCC 27853	>2000	1000
Gram positive		
*Listeria grayi* DSM 20601	250	250
*Staphylococcus aureus* ATCC 29230	1000	250

Phytopathogen bacteria		
Gram negative		
*Agrobacterium tumefaciens *DSM 30207	500	250
*Agrobacterium vitis *DSM 6583	2000	250
*Pseudomonas syringae *pv.* syringae *DSM 10604	250	500
Gram positive		
*Clavibacter michiganensis *subsp.* nebraskensis *DSM 20400	62	500

^(a)^Values were determined by the broth microdilution method.
